# HOTAIR Contributes to Stemness Acquisition of Cervical Cancer through Regulating miR-203 Interaction with ZEB1 on Epithelial-Mesenchymal Transition

**DOI:** 10.1155/2021/4190764

**Published:** 2021-09-10

**Authors:** Wenying Zhang, Jing Liu, Qiongwei Wu, Yu Liu, Chengbin Ma

**Affiliations:** Department of Gynecology, Shanghai Changning Maternity and Infant Health Hospital, Shanghai 200051, China

## Abstract

Cervical cancer stem cells contribute respond to considerable recurrence and metastasis of patients with cervical cancer. The stemness properties were partly regulated by the interaction of lncRNAs and miRNAs. HOTAIR functions as an oncogenic lncRNA. Previous research studies revealed its role in regulating stemness properties in various cancers. However, the role of HOTAIR in cervical cancer stem cells is still unknown. Here, cisplatin-resistant and serum-free cultured cells exhibited stem cells properties. Cervical cancer stem cells exhibited greater invasion and migration compared with their parental cells, which was similar to cells overexpressing HOTAIR. HOTAIR was significantly overexpressed in cervical cancer stem cells, and knockdown of HOTAIR generated statistical downregulation of stemness markers. Additionally, HOTAIR expression was negatively correlated with the level of miR-203, which was found to be an inhibitory miRNA in regulating the expressions of stemness markers. Also, miR-203 expression was negatively correlated with ZEB1. These findings suggested that HOTAIR should be a positive contributor in stemness acquisition of cervical cancer cells, and this effect should correlate with the interaction with miR-203, which can be suppressed by ZEB1.

## 1. Introduction

Despite considerable advances in screening, diagnosis, prevention, and treatment, cervical cancer is still one of the leading causes of cancer-related death in women worldwide [1, 2]. Surgery and chemoradiotherapy offer survival advantage for patients with cervical carcinoma. However, recurrence occurs in ∼35% cervical cancer patients and 90% were found within three years after the initial management. Burgeoning evidence indicated that current cancer treatments failed to eradicate a subset of cells within tumor known as cancer stem cells (CSCs); therefore, this failure allows CSCs to self-renew and provoke tumor relapse [3]. Recently, various long noncoding RNAs (lncRNAs) have been recognized crucial players in regulating CSC properties and allowing them to self-renew and promote tumor growth [4, 5]. Thus, these lncRNAs hold potential as specific targets to eliminate the CSC fraction, thereby, to eradicate cancer.

HOX transcript antisense intergenic RNA (HOTAIR) is transcribed from the antisense strand of the homeobox gene C cluster (HOXC) and recognized as an oncogenic lncRNA in various types of malignant tumors, including cervix cancer. HOTAIR recruited the PRC2 (polycomb repressive complex 2) complex to induce methylation of H3K27me3 and ultimately provoke gene silencing. Also, HOTAIR can recruit the MLL1 methyltransferase and induce H3K4me3, thereby relaxing chromatin and allowing binding of transcription machinery [6, 7]. Besides, HOTAIR is capable to sponge definite miRNAs to avoid their effect on mRNA targets. Through aforementioned approaches, HOTAIR exerts its crucial effects on proliferation, migration, and invasion in cervical cancer. Recently, HOTAIR has been reported to be elevated in CSCs derived from various malignant tumors [8]. HOTAIR overexpression is believed to be associated with the acquisition of stem cell properties, which resulted in an increased tumor growth and metastatic potential [9, 10]. Our previous data showed that HOTAIR participated in chemoresistance of cervical cancer through triggering epithelial-to-mesenchymal transition (EMT), which was considered the main factor responding for stemness acquisition by HOTAIR [11]. Actually, HOTAIR interacted to miR-203, which has been recognized as a pivotal contributor in EMT and cancer stem cell properties through suppressing the expressions of various targets. However, the role of HOTAIR and miR-203 in acquisition of cervical cancer stem cells is still unknown [12].

In present study, chemotherapeutics resistance and serum-free culture were proven to be effective to enrich cell subpopulation with stemness properties from human cervical cancer HeLa and SiHa cells. HOTAIR was statistically upregulated along with enrichment of cervical cancer stem cells, and knockdown of HOTAIR significantly downregulated the expressions of stemness markers. The HOTAIR level was negatively correlated to the expression of miR-203, which promoted EMT and regulated by ZEB1.

## 2. Materials and Methods

### 2.1. Cell Culture

The human cervical adenocarcinoma cell lines HeLa (ATCC® CRM-CCL-2^™^) and the cervical squamous carcinoma cell SiHa (ATCC® HTB-35^TM^) obtained from American Type Culture Collection (ATCC; Manassas, VA, USA) were cultured in Eagle's Minimum Essential Medium (Catalog No. 30-2003) supplemented with 10% fetal bovine serum (FBS) (st30-3302; PAN, Germany) and were maintained at 37°C in a 5% CO_2_ humidified atmosphere. Cell layers that reached 80% confluence were dispersed by 0.25% (w/v) Trypsin-0.53 mM EDTA solution. Then, cells were treated with a complete growth medium and aspirated by gently pipetting. The cell suspension was transmitted to a new culture flask at a ratio of 1 : 3 and incubated at 37°C in a 5% CO_2_ humidified atmosphere. During subcultivation, the medium was renewed 3 times per week. Cells within 3 to 5 passages were used in this experiment.

### 2.2. Enrichment of Cervical Cancer Stem Cells

HeLa and SiHa cells were trypsinized and washed twice with PBS. After that, the cells were resuspended with serum-free DMEM/F12 medium containing 2% B27, 20 ng/mL bFGF, and 20 ng/mL EGF. The cells were incubated at 37 °C with 5% CO_2_. Subculture was performed every 2–4 days.

### 2.3. qPCR Assay

Total RNA was extracted with TRIzol Reagent (Cat. No: 15596018; Invitrogen, Thermo Fisher Scientific, Wilmington, DE, USA), and its purity was determined using the NanoDrop spectrophotometer (ND-2000; Thermo Fisher Scientific, Waltham, MA, USA). The RNA samples with high purity were reversely transcribed into complementary DNAs (cDNAs) using the TAKARA PrimeScript™ RT reagent kit with gDNA Eraser (Perfect Real Time) (Cat. No: RR047A; Takara, Dalian, China) according to the manufacturer's instruction. In order to determine miRNA-203 expression, the microRNAs were purified using the SanPrep Column microRNA Extraction Kit (Cat. No: B518811; Sangon Biotech (Shanghai) Co., Ltd., China) and the first-strand cDNA synthesis was carried out using miRNA First-Strand cDNA Synthesis (Tailing Reaction) (Cat. No: B532451; Sangon Biotech (Shanghai) Co., Ltd., China) according to the manufacturer's protocol. PCR amplification was carried out in a 20 *μ*L reaction system, which contained 2 *μ*L cDNA, 10 *μ*L SYBR Premix Ex Taq II (TaKaRa, Otsu, Shiga, Japan), and 10 *μ*M of both sense and antisense primers. The expressions were calculated using the 2^−ΔΔCT^ method. Experiments were performed in triplicate. Primers used here are presented in Table 1.

### 2.4. Cell Transfection

Cell transfection was performed using Lipofectamine 2000 (Invitrogen) according to the manufacturer's protocol. In brief, the target plasmid (1 *μ*g) and Lipofectamine 2000 (3 *μ*L) were previously diluted by 100 *μ*L serum-free Opti-MEM medium (Gibco, Grand Island, NY) and then mixed up after being placed at room temperature for 5 min. Then, the mixture was added into the medium of cells that reached to 50% confluence and left for another 20 min. After being incubated for 6–8 hours, the original medium was replaced with a complete growth medium, and then, the cells were incubated for 24–48 hours. In the present study, recombinant vector pc.DNA3.1-HOTAIR was transfected into cells to overexpress HOTAIR, while cells transfected with pc.DNA3.1 were used as controls. Small interference RNA targeting HOTAIR (F: CTCCGCTTCGCAGTGGAATGG; R: TCTCGCCGCCGTCTGTAACTC) was transfected into cervical cancer cells to knockdown HOTAIR, and cells transfected with scramble siRNAs were used as controls. In order to explore the role of miR-203 in cervical cancer cells, miR-203 mimics and miR-203 inhibitor were respectively transfected into cells.

### 2.5. Cell Migration and Invasion Assay

Migration and invasion of differently treated cervical cancer cells were determined by using culture inserts of 8 *μ*m pore size (Transwell, Costar) that were placed into the wells of 24-well culture plates. For invasion assay, cell inserts were precoated with Matrigel Matrix (BD Biosciences) (100 *μ*g/well). The 1 × 10^5^ cells in serum-free medium were seeded into the upper chamber, while the lower chamber was added with 500 *μ*L of DMEM medium supplemented with 10% FBS. After being incubated at 37°C with 5% CO_2_ for 48–72 h, the cells migrated or invaded through the pores were fixed with 4% paraformaldehyde (Beyotime Biotechnology, Shanghai, China) and stained with 0.1% crystal violet (Beyotime Biotechnology, Shanghai, China) for 30 min. Images were captured, and the cells were calculated on five random fields of each insert.

### 2.6. Migration Assay

The differently treated cervical cancer cells were harvested and seeded into 6-well plates at a concentration of 1500 cells/well. Then, the cells were grown in Eagle's Minimum Essential Medium supplemented with 10% FBS until confluence. Thereafter, a vertical line was drawn at the bottom of a six‐well plate, which went through the center of the plate before seeding cells. The cells were photographed, and the wound width was quantified at 0 and 48 hours.

### 2.7. Western Blotting

The proteins of differently treated cervical cancer cells were extracted with RIPA Lysis Buffer (PP110; Protein Biotechnology Co., Ltd, China) according to the manufacturer's instruction. Then, the concentration of the proteins was determined using a BCA protein assay kit (PP202; Protein Biotechnology Co., Ltd, China). Subsequently, 30 µg proteins in each group were separated by 12% sodium dodecyl sulfate polyacrylamide gel electrophoresis (SDS-PAGE). The gels were then transferred onto a polyvinylidene fluoride (PVDF) membrane. The membrane was incubated in Tris-buffered saline containing 0.1% Tween-20 and 5% nonfat milk for 1 h. Then, the PVDF membrane was incubated overnight with primary antibodies against NANOG (ab203919; Abcam, USA), OCT4 (ab200834; Abcam, USA), CD133 (ab278053; Abcam, USA), SOX2 (ab92494; Abcam, USA), and *β*-actin (ab8227; Abcam, USA). Then, the membrane was incubated with HRP-conjugated secondary antibody (ab205718; Abcam, USA) for 2 h at room temperature. The bands of individual proteins were visualized using a chemiluminescence (ECL) kit (PP404; Protein Biotechnology Co., Ltd., China).

### 2.8. Statistical Analysis

Data were presented by mean ± standard deviation and analyzed with SPSS 21.0 (SPSS, IBM Corp, Armonk, NY). The differences between groups were analyzed with one-way analysis of variance (ANOVA) or two-tailed Student's *t*-test. The correlation of miR-203 expression with HOTAIR was analyzed with Pearson's correlation analysis. *P* < 0.05 was considered statistical significance.

## 3. Results

### 3.1. Cisplatin-Resistant Cells and Serum-Free Cultured Cells from Cervical Cancer Cell Lines Exhibit Stem-Like Properties

**Figure 1:**
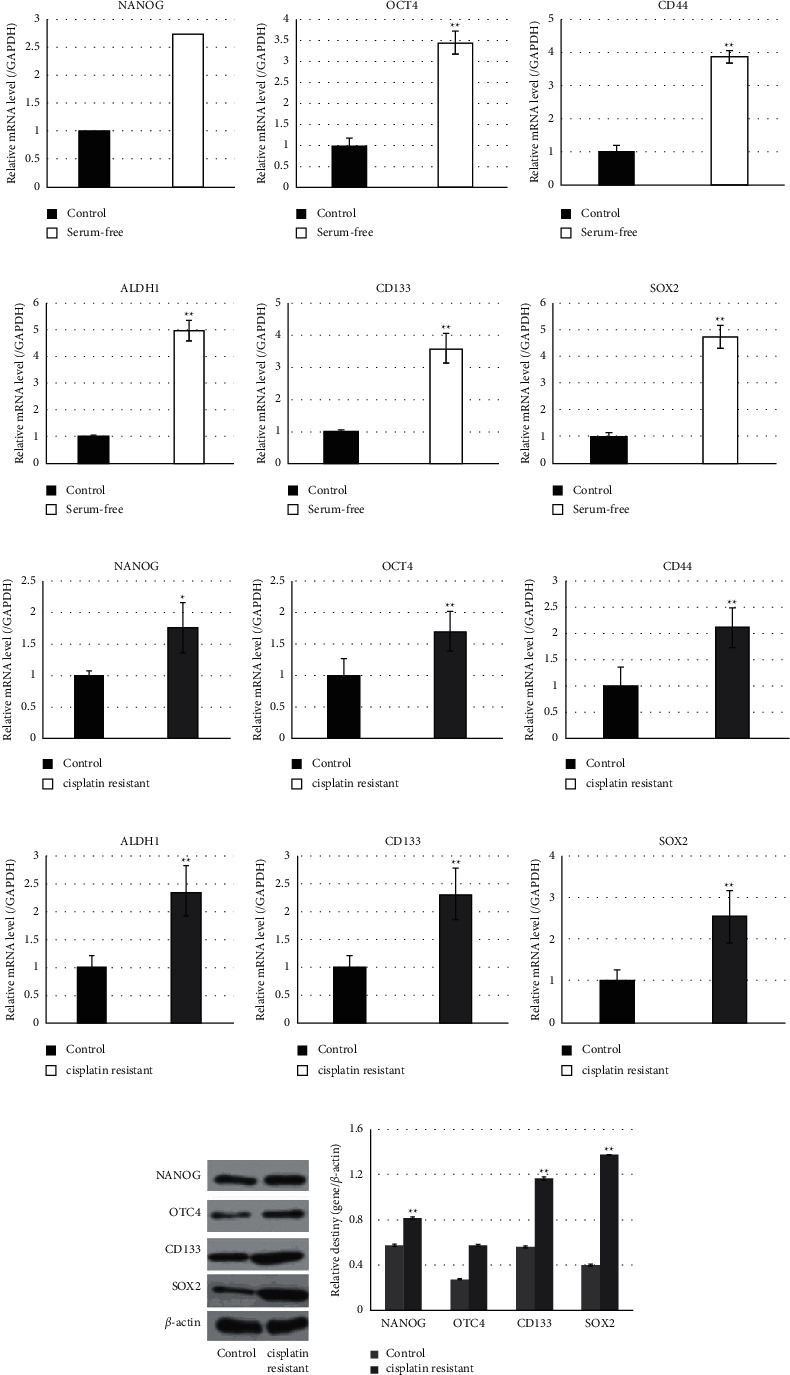
Cisplatin-resistant cells and serum-free cultured cells from cervical cancer cell lines exhibit stem-like properties. (a–f) Detection of the stem cell markers NANOG, OCT4, CD44, ALDH1, CD133, and SOX2 in HeLa cells cultured in a serum-free medium by qRT-PCR. (g–l) Detection of the stem cell markers NANOG, OCT4, CD44, ALDH1, CD133, and SOX2 in cisplatin-resistant HeLa cells by qRT-PCR. (m) Western blot analysis of the stem cell markers NANOG, OCT4, CD133, and SOX2 in cisplatin-resistant HeLa cells. (n) Statistic analysis relative destiny of (m). ^*∗*^*P* values represent significant difference based on Student's *t*-test from three replicates (^*∗*^*P*, ^*∗∗*^*P* < 0.01).

In this study, stem-like cells were enriched from two human cervical cancer cell lines, HeLa and SiHa, in presence of cisplatin and in a serum-free medium. Then, enriched cisplatin-resistant cells and serum-free cultured cells were submitted to detect the expressions of stem cell markers, including NANOG, OCT4, CD44, ALDH1, CD133, and SOX2. Data showed that the expression level of these six marker genes in serum-free cultured HeLa cells revealed a similar higher expression level of stem cell biomarkers than their parental cells (Figure 1(a)–1(f)). And, the expression level in cisplatin-resistant HeLa cells was significantly higher than that in their parental cells (Figure 1(g)–1(l)). The same results were also found in SiHa cells (data not shown). These findings suggested both cisplatin-resistant cells and serum-free cultured cells from cervical cancer cell lines exhibited stem-like properties.

### 3.2. Cervical Cancer Stem-Like Cells Hold Superior Migration and Invasion Capability

**Figure 2:**
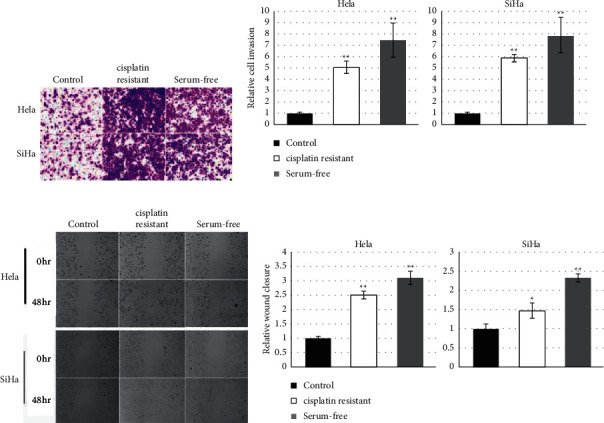
Cervical cancer stem-like cells hold superior migration and invasion capability. (a, b) Transwell assays indicated that both cisplatin-resistant and serum-free cultured cells exhibited promoted invasive ability in HeLa and SiHa cells (×100, magnifications). (c, d) The migration ability of cancer stem cells enriched after cisplatin or serum-free culture medium treatment was enhanced in HeLa and SiHa cells. ^*∗*^*P* values represent significant difference based on Student's *t*-test from three replicates (^*∗*^*P* < 0.05, ^*∗∗*^*P* < 0.01).

In this study, HeLa and SiHa cells were treated with cisplatin or cultured in a serum-free medium to enrich cervical cancer stem cells. And then, cervical cancer stem-like cells and their parental cells were submitted to analyze invasion and migration. Compared with parental HeLa and SiHa cervical cancer cells, cancer stem cells resistant against cisplatin revealed significantly more invaded cells through the pore (Figure 2(a) and 2(b)) and narrow wound closure (Figure 2(c) and 2(d)), which were inferior to the data from cancer stem cells cultured in the serum-free medium.

### 3.3. HOTAIR Promoted Migration and Invasion of Cervical Cancer Cells In Vitro

**Figure 3:**
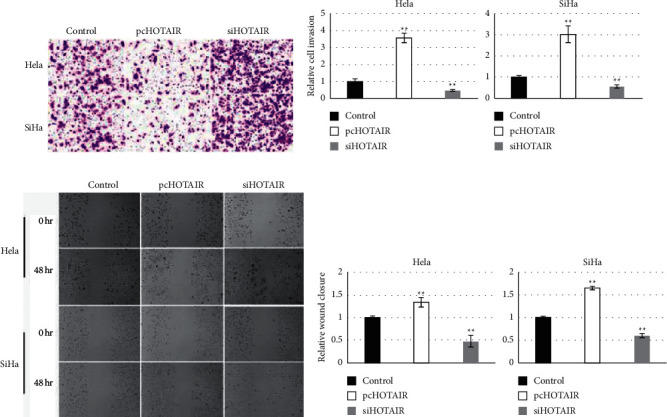
HOTAIR promoted migration and invasion of cervical cancer cells in vitro. (a, b) Transwell assays showed that HOTAIR promoted the invasion of HeLa and SiHa cells (×100, magnifications). (c, d) HOTAIR promoted the migratory ability in HeLa and SiHa cells as indicated by wound healing assay. ^*∗*^*P* values represent significant difference based on Student〙s *t*-test from three replicates (^*∗*^*P* < 0.05, ^*∗∗*^*P* < 0.01).

The role of HOTAIR in migration and invasion of cervical cancer cells was determined in vitro through both gain-of-function and loss-of-function approaches. As shown in Figure 3, the overexpression of HOTAIR resulted in statistically more invaded either HeLa or SiHa cells through the pores, while interference targeting HOTAIR revealed a significant decrease in invaded cervical cancer cells. In addition, the effect of HOTAIR on migration of both HeLa and SiHa cells were analyzed using wound closure assay. Data showed that overexpression of HOTAIR produced statistically promoted migration of both HeLa and SiHa cells, while knockdown of HOTAIR exhibited significantly lower migration of either HeLa or SiHa cells. These findings suggested that HOTAIR promoted migration and invasion of cervical cancer cells in vitro.

### 3.4. Knockdown of HOTAIR Downregulated Expression of Stem Makers in Cervical Cancer

**Figure 4:**
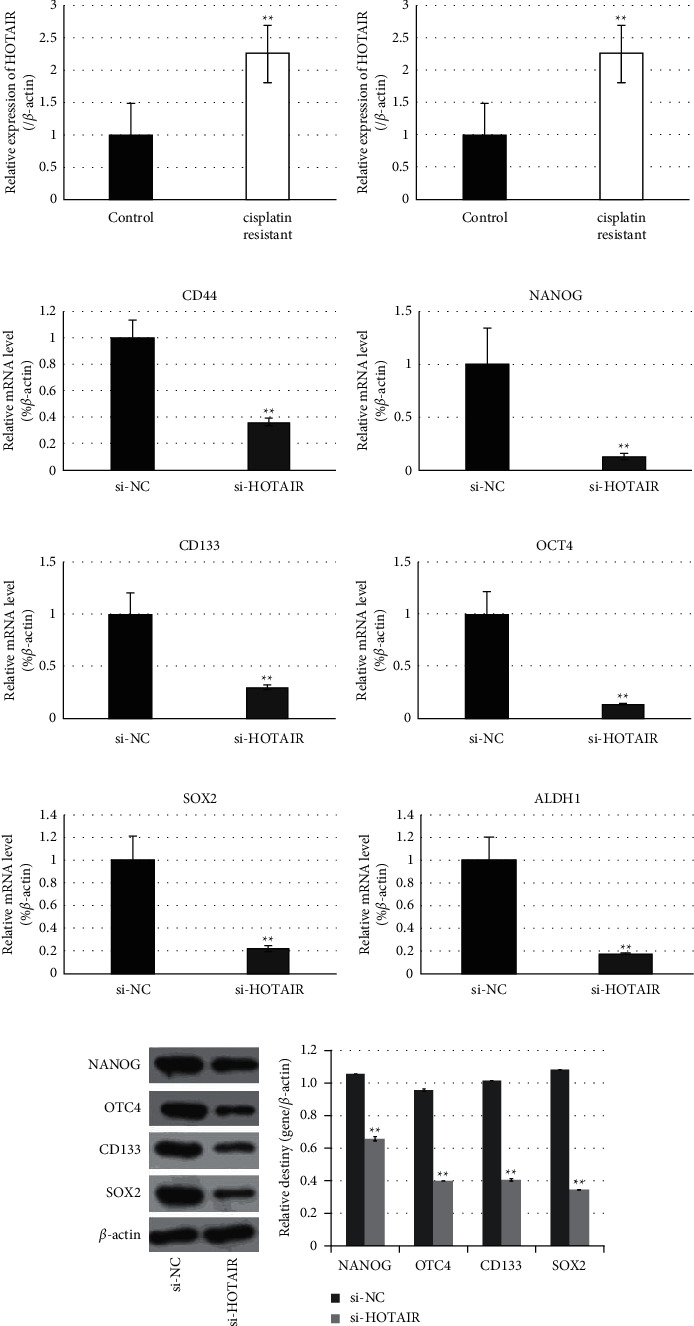
RNA interference targeting HOTAIR generated downregulation of stem cell markers. (a, b) Cancer stem cells of HeLa enriched by treatment with cisplatin (a) or culture in a serum-free medium (b) showed elevated expression of HOTAIR. (c–h) RNA interference targeting HOTAIR downregulated the stem cell markers NANOG, OCT4, CD44, ALDH1, CD133, and SOX2 in cancer stem cells of HeLa. NC, negative control. (i) Western blot analysis of the stem cell markers NANOG, OCT4, CD133, and SOX2 in cisplatin-resistant HeLa cells after RNA interference targeting HOTAIR. (g) Statistic analysis relative destiny of (i). ^*∗*^*P* values represent significant difference based on Student's *t*-test from three replicates (^*∗*^*P* < 0.05, ^*∗∗*^*P* < 0.01). miR-203 downregulated the expressions of stem markers of cervical cancer.

In order to explore whether HOTAIR participates in regulating stem properties of cervical cancer, the levels of HOTAIR in two stem-like cells and their parental cells were evaluated. Data showed that the HOTAIR level in both stem-like cells was higher than that in their parental cells (Figure 4(a) and 4(b)). Furthermore, the role of HOTAIR on stem properties of cervical cancer was explored through the loss-of-function approach. As shown in Figure 4(c) and 4(d), transfection of small interference targeting HOTAIR resulted in a statistical decrease of all six stem markers NANOG, OCT4, CD44, ALDH1, CD133, and SOX2. These findings suggested that HOTAIR should hold a positive role on stem properties of cervical cancer.

**Figure 5:**
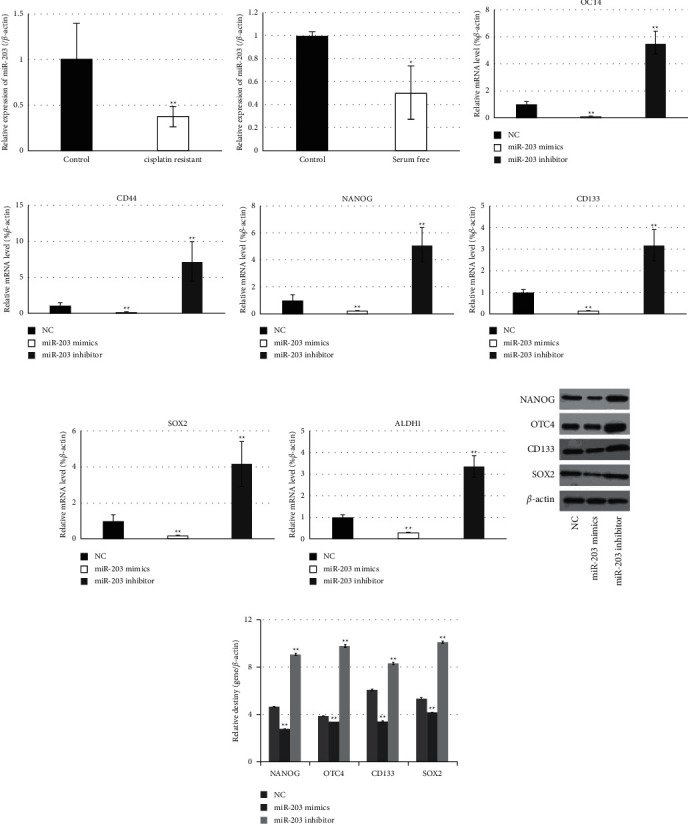
miR-203 downregulated the expressions of stem markers of cervical cancer. (a, b) Cancer stem cells of HeLa enriched by treatment with cisplatin (a) or culture in a serum-free medium (b) showed the downregulated expression of miR-203. (c–h) Expression changes of the stem markers NANOG, OCT4, CD44, ALDH1, CD133, and SOX2 in cancer stem cells of HeLa along with miR-203 alteration were detected and semiquantified by qRT-PCR. NC, negative control. (i). Western blot analysis of the stem cells markers NANOG, OCT4, CD133, and SOX2 in cisplatin-resistant HeLa cells along with miR-203 alteration. (g). Statistic analysis relative destiny of (i). ^*∗*^*P* values represent significant difference based on Student's *t*-test from three replicates (^*∗*^*P* < 0.05, ^*∗∗*^*P* < 0.01). HOTAIR interacted with miR-203 to post-transcriptionally regulate the expressions of ZEB1.

In order to explore whether miR-203 involves in regulating the stemness of cervical cancer cells, the expression level of miR-203 in cisplatin-resistant cells, serum-free cultured cells, and their parental cells were identified.  Figure 5(a) and 5(b) shows that miR-203 was statistically downregulated accompanying with the acquisition of stem properties of cervical cancer. Furthermore, the expression changes of stem markers along with miR-203 alteration were detected and semiquantified. Data showed that transfection with miR-203 mimics caused statistical downregulation in OCT4, CD44, NANOG, CD133, SOX2, and ALDH1, while cells treated with the miR-203 inhibitor revealed significant upregulation of six genes mentioned above (Figure 5(c)–5(g)). These findings indicated that miR-203 should exert a negative effect on stem properties of cervical cancer.

**Figure 6:**
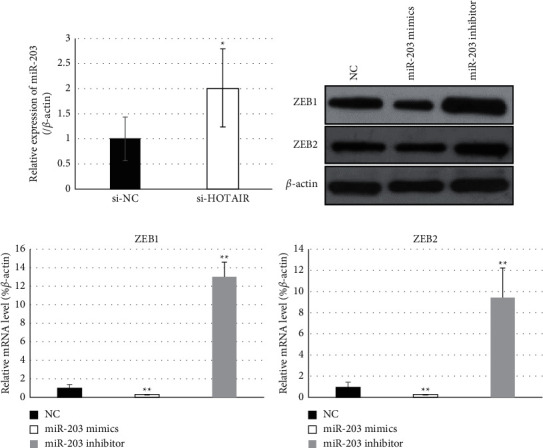
HOTAIR interacted with miR-203 to regulate the expression of ZEB1 and ZEB2. (a) Cells were treated with RNA interference targeting HOTAIR, and the miR-203 level was detected by qRT-PCR. The result indicated that miR-203 was negatively correlated with HOTAIR in HeLa cells. (b–d) Expression changes of ZEB1 and ZEB2 along with alteration of miR-203 was detected and analyzed by Western blot (b) and qPCR (c, d). NC, negative control. ^*∗*^*P* values represent significant difference based on Student's *t*-test from three replicates (^*∗*^*P* < 0.05, ^*∗∗*^*P* < 0.01).

The interaction between HOTAIR and miR-203 was explored in further study. We found that transfection with small interference RNA targeting HOTAIR resulted in a statistical increase of miR-203 compared with those normal control cells (Figure 6(a)), indicating that miR-203 was negatively correlated with HOTAIR in cervical cancer cells. Furthermore, the expression changes of ZEB1 and ZEB2 along with alteration of miR-203 were detected and analyzed (Figure 6(b)). Compared with normal control cells, transfection with miR-203 mimics generated statistical downregulation of ZEB1 and ZEB2, while transfection with the miR-203 inhibitor revealed a significant increase of their levels (Figure 6(c) and 6(d)).

## 4. Discussion

Cervical cancer is the second most common type of malignant tumors and the fourth leading cause of cancer-associated mortalities in women worldwide [6, 7]. Cervical carcinoma has a risk of considerable recurrence and metastasis following conventional therapy, leading to a high mortality. Currently, more and more evidence has confirmed that a small population of cancer stem cells may be responsible for tumor recurrence, relapse, metastasis, and resistance to conventional treatment [13]. Thus, a complete understanding of the molecular mechanisms underlying acquisition and maintaining of stemness of cervical cancer stem cells (CCSCs) is essential to provide effective methods to eradicate malignant tumors.

Cancer stem cells are a subpopulation within cancer cells, characterized by the abilities of self-renewal and differentiation into mature and specialized cancer cell types. Cancer stem cells express specific markers. However, there is currently no universal markers for isolation and identification of cancer stem cells in any type of cancer. Therefore, cancer stem cells are commonly harvested and enriched through different approaches based on its characteristics [14]. In current approaches, cell lines cultured with definite chemotherapeutics or with defined serum-free culture conditions are commonly used methods to enrich cancer stem cells from mixed populations and have been recognized effective to establish *in vitro* models for cancer stem cells expansion [15]. Thus, these two methods were employed in the present study to harvest and enrich cervical cancer stem cells from the human cervical adenocarcinoma cell lines HeLa and the cervical squamous carcinoma cell SiHa. Data showed that either cisplatin-resistant cells or serum-free cultured cells expressed statistically higher expression levels of CD133 and CD44, which have been confirmed as contributors in migration and aggregation of cancer cells and cancer development and hence have been broadly accepted as general cancer stem cell markers in many types of malignant tumors, including cervical carcinoma [13]. Additionally, cisplatin-resistant cells and serum-free cultured cells increased the expression of ALDH1, OCT4, and NANOG. Previous data have identified that the high activity of ALDH1 in a subpopulation of cervical cancer cells exhibited stemness properties with great capacity for self-renewal, high differentiation potential, and high tumorigenicity. Also, cells with high ALDH1 activity were resistant to cisplatin and increased the expression of OCT4 and NANOG, which are pluripotency markers [16]. Our data also showed that cisplatin resistance and serum-free culture significantly upregulated SOX2. Previous reports demonstrated that SOX2-positive cervical cancer cells shared all the characteristics with cancer stem cells including self-renewal, differentiation, and tumor-initiating properties [17]. And, SOX2-positive cells displayed overexpression of OCT4 and ALDH1. Therefore, the overexpression of these stemness-associated markers showed here indicated that cisplatin-resistant and serum-free cultured cervical cancer cells were cancer stem-like cells.

Cervical cancer stem cells have been recognized essential contributors for distal metastasis and recurrence of cervical carcinoma. As expected, cervical cancer stem-like cells enriched from either HeLa or SiHa cells revealed greater capability to migrate and invade *in vitro* compared with their parental cells. Interestingly, HOTAIR also promoted the migration and invasion level of cervical cancer cells, just like cervical cancer stem-like cells. Similarity of migration and invasion suggested that HOTAIR should exert a positive effect on stemness acquisition of cervical cancer cells. Actually, HOTAIR's pivotal role on stem cells of various types of malignant tumors has been reported previously [18], but the role of HOTAIR on the phenotype of cervical cancer stem cells was currently unknown. In order to identify this hypothesis, effects of HOTAIR on the expressions of stemness-associated markers was explored in present study. Data showed that treatment of cisplatin resistance and serum-free culture actually resulted in significant upregulation of HOTAIR; furthermore, knockdown of HOTAIR generated statistical downregulation of the six stemness markers mentioned above. These findings suggested that HOTAIR should be a contributor of stemness acquisition of cervical cancer.

HOTAIR regulated the stemness of cancer stem cells through various approaches, among which HOTAIR interaction with definite miRNAs was generally accepted as an important mechanism. Bioinformatics analysis and previous studies have identified that HOTAIR can interact with miR-203, which has been considered a tumor suppressive miRNA. Current reports have documented that miR-203 is a stemness-inhibiting miRNA owing to its modulation of stemness properties through regulating the expressions of various targets in different types of cancers, including SOCS3, survivin, Bmi-1, and GATA6. [19–23]. Actually, epigenetic silencing of miR-203 is required for cancer stem cell properties and epithelial-mesenchymal transition (EMT), which is a rich source of cancer stem cells [24–29]. The stemness-inhibitory role of miR-203 in cervical cancer was confirmed in the present study, which is exhibited as statistical decreased levels along with enrichment of cervical cancer stem cells and the suppressive effect on the expressions of all six stemness markers. Besides, ZEB1's ability to repress stemness-inhibiting miR-203 expression to promote tumorigenicity can be seen in previous studies [30]. It was presumed that the interaction of miR-203 with ZEB1 should contribute to stemness acquisition of cervical cancer cells.

In conclusion, both chemotherapeutics resistance and serum-free culture can be used to enrich cancer stem cells from human cervical cancer HeLa and SiHa cells. And, lncRNA HOTAIR holds positive effects for stem acquisition of cervical cancer, which should correlate to its interaction with miR-203. The EMT activator ZEB1 could regulate miR-203 expression, therefore, to maintain stem properties of cervical cancer cells.

## Figures and Tables

**Table 1:** Primer sequences in qPCR assay.

Primer Sequence (5′–3′)	
HOTAIR-F	CTCCGCTTCGCAGTGGAATGG
HOTAIR-R	TCTCGCCGCCGTCTGTAACTC
CD133-F	ACCGACTGAGACCCAACATC
CD133-R	GACCGCAGGCTAGTTTTCAC
NANOG-F	CTCGCTTCGGCAGCACA
NANOG-R	TGCTGGAGGCTGAGGTATTT
SOX2-F	TTTTGTCGGAGACGGAGAAG
SOX2-R	TTCATGTGCGCGTAACTGTC
CD44-F	TGGAGCAAACACAACCTCTG
CD44-R	TGAGTCCACTTGGCTTTCTG
OCT4-F	GAAGGATGTGGTCCGAGTGT
OCT4-R	TGAAGTGAGGGCTCCCATAG
ALDH1-F	TCCTGGTTATGGGCCTACAG
ALDH1-R	CAAGTCGGCATCAGCTAACA
HS-ACTB-F	CCTGGCACCCAGCACAAT
HS-ACTB-R	GGGCCGGACTCGTCATAC
U6-F	CTCGCTTCGGCAGCACA
miR-203-F	GCCGCAGTGGTTCTTAACAGTTCA
General reverse primer for amplifying miRNAs	AACGCTTCACGAATTTGC

## Data Availability

The data used to support the findings of this study are included within the article.
